# Effect of early treatment with polyvalent immunoglobulin on acute respiratory distress syndrome associated with SARS-CoV-2 infections (ICAR trial): study protocol for a randomized controlled trial

**DOI:** 10.1186/s13063-021-05118-7

**Published:** 2021-02-28

**Authors:** Aurélien Mazeraud, Bruno Gonçalves, Philippe Aegerter, Letizia Mancusi, Christine Rieu, Fernando Bozza, Khaoussou Sylla, Shidasp Siami, Tarek Sharshar

**Affiliations:** 1 GHU Paris Psychiatrie et neurosciences, Service de Neuroanesthésie Neuroréanimation, Paris, France; 2 Univeristé de Paris, Paris, France; 3 Instituto Estadual do Cérebro Paulo Niemeyer, Rio de Janeiro, Brasil; 4 GIRCI-IDF, Cellule Méthodologie, Paris, France et Université Paris-Saclay, UVSQ, Inserm, Équipe d’Épidémiologie respiratoire intégrative, CESP - Centre de recherche en Epidémiologie et Santé des Populations U1018 INSERM UPS UVSQ, Villejuif, France; 5 CH Sud-Essonnes, Service de Réanimation, Etampes, France

**Keywords:** Acute respiratory distress syndrome, COVID-19, SARS-CoV-2, Polyvalent immunoglobulin

## Abstract

**Background:**

As of mid-June 2020, 7,500,000 people were infected with SARS-CoV-2 worldwide and 420,000 people died, mainly from coronavirus disease 2019 (COVID-19)-related acute respiratory distress syndrome (ARDS). COVID-19-related ARDS is subject to a mortality rate of 50% and prolonged period of mechanical ventilation, with no specific pharmacological treatment currently available (Infection au nouveau Coronavirus (SARS-CoV-2), COVID-19, France et Monde. https://www.santepubliquefrance.fr/dossiers/coronavirus-covid-19). Because of its immunomodulatory action, we propose to evaluate the efficacy and safety of intravenous immunoglobulin (IVIG) administration in patients developing COVID-19-related ARDS.

**Methods:**

The trial is a phase III double-blind, randomized, multicenter, parallel group, concurrent, controlled study in hospitalized participants with COVID-19 requiring mechanical ventilation using a sequential design. Participants in the treatment group will receive infusions of polyvalent immunoglobulin for 4 consecutive days, and the placebo group will receive an equivalent volume of sodium chloride 0.9% for the same duration. The primary outcome is the number of ventilator-free days up to the 28th day. Secondary objectives are to evaluate the effect of IVIG on (1) organ failure according to the Sequential Organ Failure Assessment (SOFA) score at 14 and 28 days, (2) lung injury score at 14 and 28 days, (3) the occurrence of grade 3 or 4 adverse events of IVIG, (4) length of intensive care unit (ICU) stay, (5) length of hospital stay, (6) functional outcomes at day 90 defined by the activities of daily living and instrumental activities of the daily living scales, and (7) 90-day survival. One hundred thirty-eight subjects will be randomized in a 1:1 ratio to IVIG or placebo groups (69 in each group), considering 90% power, alpha level 0.05 (two sides), and 0.67 effect size level.

**Discussion:**

The ICAR trial investigates the effect of IVIG in COVID-19-related ARDS. We expect an increase in the survival rate and a reduction in the duration of mechanical ventilation, which is associated with significant morbidity.

**Trial registration:**

EudraCT 2020-001570-30. ClinicalTrials.gov NCT04350580. Registered on 17 April 2020

## Administrative information

**Table:** 

**Title {1}**	Value of early treatment with polyvalent immunoglobulin in the management of acute respiratory distress syndrome associated with SARS-CoV-2 infections (ICAR trial): protocol for a randomized controlled trial
**Trial registration {2a and 2b}.**	EudraCT number: 2020-001570-30 Clinicaltrials.gov id NCT04350580 on 17 April 2020
**Protocol version {3}**	Version 4.1– 19.10.2020
**Funding {4}**	This trial is funded by the Programme Hospitalier de Recherche Clinique. The Laboratoire Français du Biofranctionnement (LFB) provided the drugs at no-cost.
**Author details {5a}**	Aurélien Mazeraud, GHU Paris Psychiatrie et Neurosciences, Service de Neuroanesthésie et Neuroréanimation. Université de Paris, Paris Bruno Gonçalves, GHU Paris Psychiatrie et Neurosciences, and Paulo Niemeyer State Brain Institute, Rio de Janeiro, Brazil Philippe Aegerter, Methodology Unit, GIRCI-IdF, 75019 Paris, France, and Université Paris-Saclay, UVSQ, Univ. Paris-Sud, Inserm, Équipe d’Épidémiologie respiratoire intégrative, U1018 CESP, 94807, Villejuif, France Letizia Mancusi, GHU Paris Psychiatrie et Neurosciences Christine Rieu, GHU Paris Psychiatrie et Neurosciences Fernando Bozza, D’Or Institute for Research and Education, Rio de Janeiro, Brazil Khaoussou Sylla, GHU Paris Psychiatrie et Neurosciences Shidasp Siami, MD, PhD, CH Sud Essonnes Réanimation polyvalente Tarek Sharshar, GHU Paris Psychiatrie et neurosciences, Service de Neuroanesthésie et Neuroréanimation. Université de Paris, Paris
**Name and contact information for the trial sponsor {5b}**	Dr Sylla Khaoussou
**Role of sponsor {5c}**	The LFB did not have any role in the design of the study and collection, analysis, and interpretation of data and in writing the manuscript. GHU Paris Psychiatrie et Neurosciences was in charge of the data collection, monitoring and management.

## Introduction

### Background and rationale {6a}

Up to now, 7,500,000 people were infected with coronavirus disease 2019 (COVID-19) worldwide and 420,000 people died, mainly from acute respiratory distress syndrome (ARDS). No specific pharmacological treatment of COVID-19-related ARDS is currently available [1].

Pulmonary lesions are related to both the viral infection and inflammatory response. Patients admitted to intensive care unit (ICU) have a systemic inflammatory response, characterized by increased plasma concentrations of interleukin (IL) 2, IL 7, IL 10, granulocyte colony-stimulating factor, interferon-inducible protein 10, monocyte chemoattractant protein-1, macrophage inflammatory protein 1α, and tumor necrosis factor-alpha [2]. In the blood, the CD4 and CD8 T cells are hyperactivated even if their count is significantly reduced. This is evidenced by immunoreactive cytometric profiles for HLA-DR (CD4 3–47%) and CD38 (CD8 39–4%) or by an increase in the proportion of highly proinflammatory Th17 CCR6+ lymphocytes. Besides, CD8 T cells exhibit a highly cytotoxic profile characterized by high concentrations of cytotoxic granules (perforin+, granulysin+, or double-positive) [3].

Because of their immunomodulatory effect that may both attenuate the inflammatory response and enhance antiviral defense, we propose to evaluate the efficacy and safety of intravenous immunoglobulin (IVIG) administration in patients with COVID-19-related ARDS. IVIG modifies T cells and dendritic cell functions. Thus, they stimulate the proliferation of regulatory T cells that modulate the activity of the CD4 and CD8 T cells particularly impaired in COVID-19 [3–5]. In addition, IVIG modulates both the humoral acquired immunity, through its effects on the idiotypic network and antibody synthesis, and the innate immunity, by antigen neutralization and modulation of phagocytic cells. These effects lead to a decrease in the production of proinflammatory cytokines and complement activation, key pathogenic factors in COVID-19-related ARDS [4–7].

It should be noted that IVIG is a treatment for various autoimmune and inflammatory diseases. Both standard and polyclonal IVIG have significantly reduced mortality in patients with Kawasaki disease [8], a post-viral vasculitis. IVIG present beneficial effects in diffuse interstitial lymphocytic pneumonitis [6] and in post-influenza ARDS [9].

There were recent case reports suggesting a positive impact of IVIG in patients with COVID-19 including three cases of ARDS and one of myocarditis [10, 11]. Then, a retrospective study showed a decrease in mortality and ventilation time in mechanically ventilated patients for a COVID-19-related ARDS treated early with a high dose of IVIG [12]. No adverse events have been reported, notably of renal impairment or allergic reactions that are the most commonly reported side effects [13–17]. Two trials indicate that IVIG-related adverse events are scarce [15, 16, 18]. This promising benefit-risk balance prompted us to carry out a multicentric, placebo-controlled therapeutic trial on IVIG in COVID-19-related ARDS.

### Objectives {7}

#### Main objective

The main objective is to determine whether the administration of IVIG at a dose of 2 g/kg up to 96 h after the start of IMV, in patients with COVID-19-related ARDS, increases the number of days alive without IMV (ventilator-free days (VFD)) up to day 28 (D28) after IMV initiation.

#### Secondary objectives

Secondary objectives are to assess the impact of IVIG on the following outcomes: (1) mortality at 28 and 90 days, (2) organ failure according to the Sequential Organ Failure Assessment (SOFA) score [19] at 14 and 28 days, (3) the severity of ARD according to the lung injury score (LIS) [20] at 14 and 28 days, (4) the occurrence of grade 3 or 4 adverse events of IVIG, (5) length of ICU stay, (6) length of hospital stay, and (7) functional outcomes at 90 days defined by the activities of daily living (ADL) [21] and instrumental activities of the daily living (IADL) scales [22].

The exploratory objectives are to assess the impact of IVIG on:

- The incidence of occurrence of pulmonary embolism as IVIG might increase the risk of thromboembolic events
- The number of delirium-free days according to the Confusion Assessment Method in Intensive Care Unit (ICU) (CAM-ICU) [23], up to day 28, as systemic immune modulation might prevent delirium occurrence
- The occurrence of ICU-acquired weakness defined by a Medical research council (MRC) sum score < 48 at ICU discharge, given that it is a marker of critical illness severity [24, 25]
- The occurrence of ventilator-associated pneumonia as IVIG might have beneficial effects on ICU-acquired immunosuppression
- Immunomodulatory effects of IVIG through an in-depth study of cytokine profile, immune cell transcriptome, and lymphocyte activation

### Trial design {8}

The ICAR trial is a phase III double-blind, randomized, multicenter, parallel group, concurrent, controlled study in ICU patients who required invasive mechanical ventilation (IMV) for a moderate to severe COVID-19-related ARDS. A randomization list will be prepared in advance, according to a 1:1 ratio, balanced by randomly sized blocks and stratified by center and time of IMV at randomization: less than 12 h, between 12 and 24 h, and between 24 and 96 h. It will be delivered centrally by means of a dedicated and secured Web server.

One interim analysis is planned.

An Independent Data Monitoring Committee (IDMC) will be responsible for closely reviewing the safety and efficacy data from the interim analysis and for providing their recommendations on the continuation of the study. The IDMC will meet after 50 participants have completed the study.

## Methods: participants, interventions, and outcomes

### Study setting {9}

Patients will be enrolled in 43 participating ICUs, from 42 nationwide French hospitals.

### Eligibility criteria {10}

Inclusion criteria:

Any patient in intensive care who meet all of the following criteria:

1. Requiring IMV for less than 72 h
2. Developing moderate to severe ARDS according to Berlin classification [26]
3. Confirmed SARS-CoV-2 infection (by polymerase chain reaction (PCR))
4. Given consent by the patient, his family, or legal representative or deferred consent (emergency clause)
5. Being affiliated to a social security scheme (or exemption from affiliation)

Exclusion criteria (any of the following):

- Allergy to polyvalent immunoglobulins
- Pregnancy or patient under 18 years of age
- Known immunoglobulin A deficiency
- Patient with acute renal failure on admission defined by a creatinine 3 times higher than baseline or creatinine > 354 μmol/L or a diuresis of less than 0.3 mL/kg for 24 h or anuria for 12 h
- Participation in another interventional trial

### Who will take informed consent? {26a}

The informed consent will be obtained in the inclusion visit carried out by a physician who is part of the research team, in each participating center. Consent could be obtained from the patient before the need of mechanical ventilation, or from a relative if the patient is unable to consent, or using the emergency clause according to French Law if no relative could be present within 24 h. If the patient was unable to consent, a pursuit consent will be sought as soon as the patient is able to give informed consent.

### Additional consent provisions for collection and use of participant data and biological specimens {26b}

A complementary consent following the same design will be sought for the biological data collection. Blood samples will be collected at D1, D3, D5, D7, D14, D21, and D28. The collections will be kept in the laboratory of the GHU Paris Biological Resource Centre for 6 months at − 80 °C.

## Interventions

### Explanation for the choice of comparators {6b}

Since there is no specific pharmacological treatment available yet for COVID-19-related ARDS, the placebo was chosen as a comparator of the IVIG therapy.

### Intervention description {11a}

Patients in the intervention group will receive an infusion of 2 g/kg of IVIG.

The first administration of IVIG should start before the end of the 96th hour after onset of mechanical ventilation.

We will administer CLAIRYG® (Laboratoire Français du biofonctionnement, Les Ulis; France) as it is a sucrose, glucose-free IVIG preparation containing low dose of maltose that reduces the risk for renal failure. The adverse event risks associated with the administration of IVIG will be lowered by an administration of four perfusions of 0.5 g/kg of IVIG each, over a period of at least 8 h. We suggested to the participating centers to infuse IVIG over a 24-h-long period rather than 8 h, if the drug supply and nurse organization could allow it, to increase the tolerance of the treatment.

Patients in the placebo group will receive an equivalent volume of sodium chloride 0.9% (10 mL/kg), over the same period.

### Criteria for discontinuing or modifying allocated interventions {11b}

Any subject can discontinue his participation in the trial at any time for any reason. The investigator may discontinue temporarily or permanently a subject’s participation in the trial for any reason concerning his safety or for his best interests. In the event of premature termination of the research, or withdrawal of consent, data collected prior to the premature termination may be used. The reasons for discontinuing participation in the research will be registered in the participant’s file.

### Strategies to improve adherence to interventions {11c}

The follow-up of the administration of IVIG and placebo vial follow-up will be reported in the regulatory medicinal products derived from human blood follow-up sheet in each center and sealed in an envelope put in the medical folder.

### Relevant concomitant care permitted or prohibited during the trial {11d}

Participating centers are following the French clinical guidelines for the management of ARDS [27].

#### Invasive mechanical ventilation policy

Timing of patient intubation to initiate ventilatory support is left to the discretion of the physician in charge of the patient. We recommend targeting tidal volume < 6 mL/kg ideal body weight. As no clear consensus emerges concerning PEEP optimization, it was also left to the discretion of the physicians.

#### Weaning from ventilation

Weaning strategies are also left to the discretion of the attending physician. The start of the weaning procedure will be based on the following consensual criteria: adequate patient responsiveness and cooperation, appropriate cough reflex, and oxygenation saturation > 90% with PaO2 to FiO2 ratio > 200 mmHg at FiO2 ≤ 0.5. Extubation will be performed after a spontaneous breathing trial is considered successful, i.e., in the absence of an increase of respiratory rate above 35 per minute or by 50% from baseline, increase in heart rate or systolic blood pressure of more than 20%, or occurrence of agitation, depressed mental status, or diaphoresis. The need for tracheostomy is left to the discretion of the attending physician.

#### Other standard procedures

Sedation, neuromuscular blocker, need for prone position, or extracorporeal life support will be left to the discretion of the attending physician. It has to be noted that levels of sedation of curarization are targeted and monitored every 4 h with help of the Richmond Agitation Sedation Scale (RASS) and Train of Four in each participating ICU.

Thrombosis prophylaxis is indicated for all patients who are not already treated with anticoagulants. In patients with suspected pulmonary embolism, curative anticoagulation is strongly recommended even without CT-scan confirmation. Fluid management will be left to the discretion of the attending physician. A diet will be started as soon as possible after ICU admission through feeding gastric tube targeting 20–25 kcal/kg/day.

### Provisions for post-trial care {30}

Any adverse event will be monitored until its complete resolution (stabilization at a level deemed acceptable by the investigator or return to the previous state) even if the participant has left the research.

### Outcomes {12}

#### Primary outcome

The primary endpoint is the number of ventilator-free days at D28, which is, by definition, the number of days the participant was alive and free from IMV from the day of randomization which is day 0 to day 28. The score is calculated by the sum of the number of days the patient did not receive IMV; but in case of death before D28, the score is 0. This is a validated and commonly used primary endpoint in trials on ARDS [28].

In case of multiple invasive mechanical ventilation periods, only the last extubation will be considered free of IMV [29].

#### Secondary outcomes

Secondary outcomes are vital status at day 28 and day 90, SOFA score at days 14 and 28, lung injury score at days 14 and 28, the occurrence of grade 3 or 4 side effects, length of ICU stay, length of hospital stay, and ADL and IADL at day 90.

The other exploratory outcomes are:

- Pulmonary embolism, proven by computed tomography (CT) pulmonary angiogram, at days 28 and 90
- The number of delirium-free days according to the CAM-ICU at day 28
- The occurrence of ICU-acquired weakness defined by a MRC sum score < 48 at ICU discharge, day 28, and day 90
- The occurrence of ventilator-associated pneumonia defined by a positive microbiological sample after 48 h of mechanical ventilation, at day 28 and day 90
- Immunomodulatory effects of IVIG through an in-depth study of cytokine profile, immune cell transcriptome, and lymphocyte activation, at days 1, 7, 14, 21, and 28

### Participant timeline {13}

Participant timeline is presented in Table 1

**Table 1:** Research timeline for each participant

Timepoint	D0	D1	D2	D3	D4	D5	D6	D7	D14	D15–20	D21	D22–27	D28	D90
Consent collection	**x**													
Pursuit consent collection		**x**	**x**	**x**	**x**	**x**	**x**	**x**	**x**	**x**	**x**	**x**	**x**	
Demographics, medical history, disease characteristics	**x**													
Administration of IVIG or placebo therapy		**x**	**x**	**x**	**x**									
Main outcome measurement		**x**	**x**	**x**	**x**	**x**	**x**	**x**	**x**	**x**	**x**	**x**	**x**	
Collection of clinical data	**x**	**x**	**x**	**x**	**x**	**x**	**x**	**x**	**x**	**x**	**x**	**x**	**x**	
Complete blood count, blood gas, creatinine	**x**	**x**						**x**	**x**		**x**		**x**	
Leukocytosis, C-reactive protein, biobank collection	**x**	**x**						**x**			**x**			
SOFA score	**x**	**x**	**x**	**x**	**x**	**x**	**x**	**x**	**x**	**x**	**x**	**x**	**x**	
Adverse events	**x**	**x**	**x**	**x**	**x**	**x**	**x**	**x**	**x**	**x**	**x**	**x**	**x**	**x**
Final assessment of main outcome													**x**	
Final assessment of secondary outcomes													**x**	**x**

.

### Sample size {14}

We hypothesized that the number of days without IMV is 10 days in the placebo group and 15 days in the experimental group, with a standard deviation of 6 days for discharged alive patients, considering mortality rates of 50% and 40% in the placebo and experimental groups respectively [30, 31]. The number of days without IMV in the placebo group is (50% × 10 d) + (50% × 0 d) or 5 days on average, and following the same calculation for the experimental group of (60% × 15 d) + (40% × 0 d) or 9 days.

A 5-day reduction in IMV duration is a reasonable and valuable clinical objective. Therefore, a median value of 5 days without ventilation in the placebo group versus 9 in the experimental group is assumed, and the 6-day standard deviation is assumed to be stable (effect size = 0.67). Given the fact that this criterion presents an inflation of zeros and that its distribution cannot be considered gaussian nor symmetric, the calculation of the sample size considered the asymptotic relative efficiency of a Wilcoxon rank-sum test vs a *t*-test. For a 90% power and a two-sided 5% alpha risk level, the number of subjects to be included is 138 patients, 69 in each arm.

### Recruitment {15}

Each clinical center involved in the ICAR trial was chosen based on its ability to include patients presenting COVID-19-related ARDS screened for the trial. We plan to include 0.3 patient per center per month until April 1 of 2021.

**Table:** 

Number of patients to be included	138
Number of centers	43
Number of months	12
Number of patients per month per center	0.3

## Assignment of interventions: allocation

### Sequence generation {16a}

Randomization lists will be prepared in advance by an independent statistician, according to a 1:1 ratio, for each stratum as defined by a combination of center and time of IMV at randomization (less than 12 h, between 12 and 24 h, and between 24 and 96 h), and balanced by randomly sized blocks. Lists will be verified according to the sponsor procedures then uploaded in a dedicated server.

### Concealment mechanism {16b}

The randomization will be held in an online platform for data entry, which ensures allocation concealment by not releasing the randomization code until the recruitment is completed. After screening for inclusion and exclusion criteria, and obtaining consent, each center’s pharmacy will be informed by email of the allocated treatment and by logging onto the access-secured dedicated randomization website. Investigators remain blinded.

### Implementation {16c}

The REDCap platform will implement the randomization list and allows to send emails to the participant’s and the promoter’s pharmacy.

## Assignment of interventions: blinding

### Who will be blinded {17a}

Both trial participants, care providers, and outcome assessors will be blinded to patients’ assignment to one of the trial groups. The blinding will be prepared by the hospital pharmacy of each establishment using opaque sleeves to hide the product and providing opaque tubules. Nurses in charge will not be blinded to the study as they receive and prepare the product before their infusion.

### Procedure for unblinding if needed {17b}

Unblinding may be requested at any time and for any reason considered legitimate by the investigating physician and proceeded by calling the Délégation à la Recherche Clinique et à l’Innovation (Delegation for Clinical Research and Innovation) or the Pharmacy of Centre Hospitalier Sainte-Anne at the phone numbers given to each center in the study protocol.

## Data collection and management

### Plans for assessment and collection of outcomes {18a}

Assessments will be done daily throughout the ICU stay until D28. If the patient has been discharged from the ICU, a visit will be made at D14, D21, and D28 to collect primary and secondary outcome data. An electronic case report file will be available to the research team of each institution, on an online platform (Research Electronic Data Capture (REDCap), Vanderbilt University) [32, 33]. Activities of daily living and instrumental activities of daily living will be collected at day 90 by telephone. Study data will be collected and managed using REDCap electronic data capture tools hosted at Centre Hospitalier Sainte-Anne. REDCap is a secure, web-based software platform designed to support data capture for research studies, providing (1) an intuitive interface for validated data capture, (2) audit trails for tracking data manipulation and export procedures, (3) automated export procedures for seamless data downloads to common statistical packages, and (4) procedures for data integration and interoperability with external sources.

Screening and eligibility data (day 0)

- Patient’s initials, gender, date of birth
- Verification of inclusion and exclusion criteria
  - Mechanical ventilation initiation time
  - PaO_2_/FiO_2_ value
  - Positive end-expiratory pressure (PEEP) value
  - Chest X-ray or lungs CT scan
  - Specimen positive for SARS-CoV-2 in PCR
  - Informed consent or emergency clause
  - Creatininemia and diuresis

Baseline data (D0)

The following data will be recorded at the baseline visit:

- Weight (measured with a weighing scale) in kilograms
- Height in centimeters
- COVID-19 characteristics, symptom onset, severity at pulmonary CT, previous treatment of COVID-19 with antiviral, corticosteroids, interleukin inhibitors, antibiotics, and hydroxychloroquine
- Pulmonary embolism on chest CT angiogram, when available
- ICU admission and invasive mechanical ventilation initiation date and time
- Simplified Acute Physiology Score (SAPS) 2 [34] at ICU admission

Daily follow-up D0–D28

- Vital status, extubation, re-intubation, tracheostomy, ICU discharge
- Supportive treatment administered: continuous intravenous sedation, neuromuscular blocker, prone position initiated in the last 24 h, nitric oxide, almitrine, extracorporeal life-sustaining support
- Respiratory variables: tidal volume, plateau pressure, compliance, PaO_2_/FiO_2_
- Weaning trial: spontaneous breathing trial or T-tube trial
- COVID-19 treatment: antiviral, corticosteroids, interleukin inhibitors, antibiotics, and hydroxychloroquine
- Biological tests: leukocyte and lymphocyte count, platelet count, fibrinogen, D-Dimer, procalcitonin, and C-reactive protein.
- Radiological score defined as the sum of quadrants with opacities
- SOFA score [19] and Kidney Disease: Improving Global Outcomes score (KDIGO) [35]
- CAM-ICU [23]
- IVIG adverse event occurrence[36]:
- Manifestations of cutaneous hypersensitivity
- Occurrence of hypersensitivity manifestation with hypotension during IVIG infusion (defined as a mean blood pressure of less than 65 mmHg for 30 min, after correction for hypovolemia)
- Doppler ultrasound evidence of deep venous thrombosis, when performed
- Existence of a pulmonary embolism proven by CT scan
- Transfusion-associated lung injury
- Aseptic meningitis defined by a clinically objectified meningeal syndrome upon awakening
- Hemolytic anemia (defined as hemoglobin less than 8 g/dL, unmeasurable haptoglobin, and a positive direct Coombs test)

D28 and D90 follow-up

- Days on mechanical ventilation (considering the first 28 days after randomization)
- Vital status and date of death (for patients who died)
- Days on tracheostomy, if realized
- ICU complications: catheter-related infection, number of episodes of ventilator-associated pneumonia (VAP), digestive hemorrhage, pressure sores (> grade 2), confusion according to the CAM-ICU [23], focal neurological deficit, toxidermia
- Functional status: MRC score at discharge [25], ADL value [21], IADL value [22]

### Plans to promote participant retention and complete follow-up {18b}

The necessary data for the assessment of the primary endpoint are systematically collected in routine care until discharge from ICU. If the patient is discharged from the hospital before D28, a telephone interview will be conducted, and the patient (or relatives) will be informed beforehand. A telephone interview will allow us to complete mandatory information such as the vital status and ventilatory status for the assessment of the main and secondary outcomes.

### Data management {19}

Trial data will be collected in the electronic case report form by the investigator or collaborators.

Source documents, being defined as any original document or object making it possible to prove the existence or accuracy of a data or fact recorded during the research, will be kept according to the regulations in force, by the investigator or by the hospital.

### Confidentiality {27}

During or at the end of the research, the data transmitted to the sponsor by the investigators (or any other specialized contributors) will be rendered non-identifiable. Under no circumstances should the names or addresses of the participants be exposed. The sponsor will ensure that each person who is subject to the research has given his or her consent for access to individual data concerning him or her that are strictly necessary for the research.

### Plans for collection, laboratory evaluation, and storage of biological specimens for genetic or molecular analysis in this trial/future use {33}

Blood samples will be collected at D1, D3, D5, D7, D14, D21, and D28 for storage and further analysis for 24 patients from predefined centers.

During the research, the collections will be kept in the laboratory of the GHU Paris Biological Resource Centre, for a period of 6 months, at − 80 °C.

### Statistical methods

A Consolidated Standards Of Reporting Trials (CONSORT) flow diagram will illustrate patient progression through the trial from initial screening for eligibility to completion of the primary outcome assessment (D28) and follow-up (D90). The number (with reasons) of losses to follow-up (28 days for patients discharged before 28 days and 90-day visit) will be summarized by treatment arm.

Full details of the statistical analysis will be detailed in a separate statistical analysis plan (SAP) which will be drafted early in the trial and finalized prior to the interim analysis data lock.

### Statistical methods for primary and secondary outcomes {20a}

#### General consideration

Continuous variables will be summarized by median and interquartile range for asymmetric distributions or else by mean and standard deviation. Categorical variables will be summarized by the proportions with their 95% confidence intervals.

#### Trial medication

The trial medication will be summarized by treatment arm. The number of patients with doses deviating from the protocol will be presented by treatment arm.

#### Primary endpoint

The Wilcoxon rank-sum test stratified by center and IMV duration will be used for the primary analysis of the principal endpoint.

The hypothesis of equality of treatment arms with respect to VFD will be tested at a two-sided significance level of 0.05 adjusted for interim analysis (see the “Interim analysis {21b}” section).

The effect of the treatment will be explored through supporting analysis on the primary endpoint and its components (mortality and duration of ventilation).

Descriptive analyses of the VFD and of ventilation time will be performed showing medians and the interquartile ranges.

#### Secondary endpoints for efficacy

As recommended by Yehya et al. [29], data will also be analyzed as time to event censored at D28, within a competing risk framework, where extubation is the main event and death before extubation a competing one. Time to each event, i.e., subdistribution hazards, will be modeled by a Fine and Gray model, with the treatment arm included as a covariate and center as a stratum.

In addition, the effect size and number needed to treat (NNT) will be computed.

The 28- and 90-day overall survival probability will be estimated by the Kaplan-Meier method. If the assumptions for appropriate use of the Cox proportional hazards regression model will be respected, in particular:

- Independence of survival times between distinct individuals in the sample
- A multiplicative relationship between the predictors and the hazard
- A constant hazard ratio over time

a comparison of the treatment arms will be performed with the Cox model by estimating the hazard ratio with 95% confidence interval, with treatment, participant’s risk factors (age, sex, and body mass index) at baseline as the model term. Center will be included as a covariate in this model.

For mortality at 28 and 90 days, the effect size and number needed to treat (NNT) will be computed.

The other efficacy outcome, such as:

- SOFA score [19] (presented as percentage variation from the baseline score at 14 and 28 days)
- Lung injury score [20]
- ADL [21] and IADL [22] score at 28 and 90 days will be presented as medians and interquartile ranges. According to their distribution, a Student or Mann-Whitney test will be performed for the treatment arm comparisons. For ADL and IADL grouped score, the chi-square test will be used.

Finally, the length of ICU stay (in days) and length of hospital stay up to the 90th day will be analyzed according to the time to discharge using the log-rank test. The Kaplan-Meier curves will be presented by treatment. Other multi-state models can be used to explore secondary endpoints.

## Methods for additional analyses {20b}

### Subgroup analyses

Subgroup analyses will be conducted for the predefined stratification factors for the study. Additional exploratory subgroup analysis will be conducted using the baseline clinical characteristics and laboratory parameters. A subgroup analysis by age, with threshold above or equal to 65 years, in the subgroup of patients alive at day 7, will be performed.

The details will be prospectively specified in the SAP.

### Covariates

For efficacy analyses, important prognostic factors that need adjustment will be specified in the SAP.

## Analysis population and missing data {20c}

### Populations for analyses

For purposes of analysis, the analysis sets are defined in Table 2

**Table 2:** Populations for analysis

Population (analysis set)	Description
Intent-to-treat (ITT) population	The ITT population will include all randomized participants. The ITT participants will be analyzed according to randomized treatment, irrespective of the actual treatment received. All efficacy analyses will be performed using the ITT population.
Modified intent-to-treat (mITT) population	The mITT population will include all randomized participants. The ITT participants will be analyzed according to randomized treatment and who have been given at least one dose of treatment. The mITT population will be used for supportive analyses of the efficacy measurements.
Per protocol (PP) population	The PP population will include all participants in the ITT population with no major protocol deviations that may significantly impact data integrity or patient safety. The PP population will be used for supportive analyses of the efficacy measurements.
Safety population (SP)	The SP will include all randomized participants who have been given at least one dose of treatment (IVIG or placebo). The SP will be analyzed according to the actual treatment received. This set will be used for the safety analyses.

.

### Safety parameters

All safety analyses will be performed on the safety population.

Safety and tolerability will be assessed by clinical safety laboratory measurements, physical examinations, vital signs, concomitant medications, cumulative incidence of adverse events, and severe adverse events.

### Adverse events

Adverse events will be coded using the MedDRA coding dictionary.

The number and percentage of patients with severe or not, related or not, adverse events will be summarized.

The number of deaths due to an adverse event and study discontinuation due to an adverse event will be summarized.

### Missing data

Patients discharged from the hospital before day 28 after randomization will be interviewed in order to check their vital, hospital (i.e., new hospital, ICU admission), and respiratory status (i.e., mechanically ventilated or not).

Given the severity of COVID-19-related ARDS, it is expected that the number of patients lost to follow-up before day 28 will be minimum.

In the primary analysis, for these patients, the VFD will be assumed equal to 0.

### Statistical software

All statistical analyses will be conducted using SPSS, version 26 (IBM Corp., Armonk, NY, USA).

## Interim analysis {21b}

One formal interim statistical analysis will be carried out when 50 (25 participants in the IVIG arm and 25 participants in the placebo arm) have completed the D28 assessment.

The purpose of the first analysis will be to assess the futility of IVIG based on the results on change in VFDs at D28. The following futility criterion will be used for this interim analysis: If the difference in the VFDs is less than 3-day improvement between both treatment arms, the benefit of IVIG treatment is not expected. For a final decision to stop the study for futility, the results on other endpoints will be considered as well.

For the primary objective (VFDs) to account for multiple testing due to the interim analysis, an adjustment for type I error alpha will be applied using the O’Brien-Fleming spending function, which would expend two-sided alpha = 0.003 at the first interim analysis (critical value = ±3.6128) and leave nominal two-sided alpha of 0.0497 for the final analysis (critical value = ±1.9601).

The details of the interim analysis will be included in the statistical analysis plan.

An Independent Data Monitoring Committee (IDMC) will be responsible for closely reviewing the safety and efficacy data from the interim analysis and for providing their recommendations on continuation of the study, including the necessity of subsequent interim analysis.

## Plans to give access to the full protocol, participant-level data, and statistical code {31c}

Access to full protocol, participant-level data, and statistical analysis will be granted upon request sent to the corresponding author of the trial. No later than 1 year after the collection of the 1-year post-randomization interviews, we will deliver a completely deidentified data set to an appropriate data archive for sharing purposes.

## Oversight and monitoring

### Composition of the coordinating center and trial steering committee {5d}

A Steering Committee is formed by the scientific manager Professor Tarek Sharshar and composed of Dr. Aurélien Mazeraud and Professor Michel Wolff. The committee will meet to evaluate the trial progress. They will advise the sponsor on the trial discontinuation or prolongation to adapt to the pandemic progression.

A Critical Events Validation Committee is formed by Dr. Mazeraud, Dr. Sharshar, Dr. Schimpf, Dr. Daniel, Dr. Legouy, and Dr. Wolff.

Its responsibility will be to validate the evaluation of the primary endpoint and secondary endpoints subject to subjective interpretation: ventilator-associated lung disease.

Methods of operation: Physical meeting or teleworking sessions 2 times during the trial. The first meeting will be held 2 months after the start of the trial and the second meeting at the end of the inclusions.

### Composition of the data monitoring committee, its role, and reporting structure {21a}

The sponsor will ensure the safety and respect of those who have agreed to participate in the research. The sponsor will establish a quality assurance system to monitor the conduct of the research in the investigative centers.

To this end, the sponsor will appoint clinical research associates and realize regular follow-up visits for each research site after the opening visits. The centralized monitoring will be performed distantly between the center’s research associates and the sponsor’s research assistant. Demographic characteristics, inclusion criterion, main outcome, and informed written consent collection will be systematically monitored for all participants. The completeness of severe adverse event reports will also be monitored.

A clinical research associate appointed by the sponsor will ensure the proper conduct of the research, the collection of data generated in writing, their documentation, recording, and reporting, in accordance with Good Clinical Practice as well as the legislative and regulatory provisions in force.

The IDMC will be composed of Professor Raphael Porcher, Professor Antoine Roquilly, Dr. Guillaume Turc, and Dr. Franck Verdonk. They will conduct the interim analysis and provide recommendations concerning the continuation, interruption, and possible next interim analysis concerning the study.

### Adverse event reporting and harms {22}

The clinical-biological parameters collected daily will allow the identification of the occurrence of adverse events. For any participant, from the signing of the informed consent, adverse events, whether serious or not (spontaneously reported by the participants or observed by the investigators, or consisting of clinical alterations, or an abnormal laboratory or radiology result), will be recorded in the case report form.

If possible, the investigator will establish a diagnosis of the adverse event based on signs, symptoms, and/or other clinical information. Then, only the diagnosis will be documented as an adverse event and not the individual symptoms/signs.

The investigator will assess for each adverse event its severity and report all serious and non-serious adverse events in the electronic case report form. The investigator will also assess the causal relationship of the serious adverse events to the trial intervention.

The method used by the investigator, based on the World Health Organization (WHO Uppsala Monitoring Centre) method, is based on the following 4 causality terms:

- Certain
- Probable/plausible
- Possible
- Unlikely (not excluded)

The investigator should immediately notify the sponsor of serious adverse events:

- From the date of initiation of treatment
- For the duration of the participant’s follow-up, as provided for by the research
- Up to 28 days after taking IVIG, following the completion of treatment with the participant’s investigational drug

The initial notification of a serious adverse event to the sponsor will be followed promptly by detailed written follow-up reports to monitor the progress of the case on a vigilant basis or to supplement the information.

The investigator will, as far as possible, provide the sponsor with any documents that may be useful (medical reports, biological results, results of further investigations). These documents should be made anonymous. Also, they must be supplemented by the following information: acronym of the research, number, and initials of the participant.

Any adverse event will be followed up until its complete resolution (stabilization at a level deemed acceptable by the investigator or return to the previous state) even if the participant has left the research.

The sponsor will inform the competent authority and the Committee for the Protection of Persons without delay of the new facts and, where appropriate, of the measures taken, from the day on which it becomes aware of them.

Following the initial report of a new development, the sponsor shall send to the competent authorities, in the form of a follow-up report on the new development, any relevant additional information relating to that new development within a maximum of 8 days from the time when it becomes available to him.

Any urgent safety measures shall be followed by an application for a substantial amendment to be submitted for authorization to the Agence Nationale de Sécurité du Médicament within 15 days.

### Frequency and plans for auditing trial conduct {23}

The investigators undertake to accept quality assurance audits performed by the sponsor as well as inspections by the competent authorities. All data, documents, and reports may be the subject of regulatory audits and inspections without prejudice to medical confidentiality.

An audit may be carried out at any time by people commissioned by the sponsor and independent of the persons responsible for the research. Its purpose is to ensure the quality of the research, the validity of its results, and compliance with the law and regulations in force.

The people conducting and monitoring the research agree to comply with the requirements of the sponsor and the competent authority concerning an audit or inspection of the research.

The audit may apply to all stages of the research, from the development of the protocol to the publication of results.

### Plans for communicating important protocol amendments to relevant parties (e.g., trial participants, ethical committees) {25}

Any substantial amendment to the protocol made by the coordinating investigator should be forwarded to the sponsor for approval. Following this agreement, the sponsor must obtain a favorable opinion from the Ethics Committee and authorization from the Agence Nationale de Sécurité du Médicament within their respective areas of competence prior to its implementation.

The information note and the consent form may be revised if necessary, in particular in the event of a substantial change in the research or the occurrence of adverse effects.

### Dissemination plans {31a}

The final report of the research involving the human subject, mentioned in Article R1123-67 of the Public Health Code, is drawn up and signed by the sponsor and the investigator. A summary of the report drawn up in accordance with the competent authority’s reference plan must be sent to the competent authority within 1 year, after the end of the research, corresponding to the end of the participation of the last person who takes part in the research.

Also, as the Trial is supported by the Réseau Recherche de la Société Française d’Anesthésie Réanimation, an oral communication will be realized at the SFAR congress.

## Discussion

ARDS related to COVID-19 is associated with an exceedingly high mortality and morbidity rate [31]. If efficient on survival and duration mechanical ventilation, a therapy could then increase the availability of ventilators and ICU beds to limit the shortage observed during the SARS-CoV-2 pandemic. The ICAR is the only randomized controlled trial assessing IVIG in COVID-19-related moderate to severe ARDS patients. It will provide high-quality evidence for demonstrating or disproved a benefit from IVIG in this condition.

We focused on the late phase of COVID-19 when patients have developed moderate to severe ARDS. One may argue that, if given at an earlier phase, the IVIG would prevent the evolution of COVID-19 towards ARDS. However, we thought targeting a less severe but wider population would not be feasible because of IVIG availability and cost.

The ICAR design, as a randomized, controlled, double-blind trial, will limit to the maximum the biases. Nurses attending patients will not be blinded to the arm allocation, but the researchers and physicians evaluating the main and secondary outcomes will be. The duration of mechanical ventilation depends on various factors, including management of sedation, curarization, and weaning procedure. The double-blind design and randomization stratification on centers along with the standardization of care will limit discrepancy between the two therapeutic groups. In addition, the main confounding factors that might impact the outcomes will be collected, including age, D-Dimer level, compliance, and time from first symptoms to intubation.

If our trial does not show benefits of IVIG and as it increases cost and is associated with serious adverse effects, benefit-risk balance will not support its use in COVID-19-related ARDS. Conversely, if it does show beneficial effects, the use of IVIG in COVID-19-related ARDS could be rapidly spread and will increase the ICU bed availability and reduce the burden of the SARS-CoV-2 epidemic.

## Trial status

ICAR trial protocol version 3.1, from 11 June 2020.

Recruitment was initiated on 10 April 2020, with the initial expected end of recruitment on 10 July 2020. Fifty-seven patients are included in the trial. Recruitment is still ongoing. An amendment is ongoing to extend the inclusion period to April 2021.

## Abbreviations

SARS-CoV-2: Severe acute respiratory distress coronavirus type 2
ICAR: Intravenous immunoglobulin in COVID-19-related ARDS
COVID-19: Coronavirus disease 2019
ARDS: Acute respiratory distress syndrome
IVIG: Intravenous immunoglobulin
ICU: Intensive care unit
VFD: Ventilator-free days
SOFA: Sequential Organ Failure Assessment
ADL: Activities of daily living
IADL: Instrumental activities of daily living
ICU: Intensive care unit
D: Day
IL: Interleukin
CD: Cluster of differentiation
HLA-DR: Human leukocyte antigen DR type
CCR: C-C chemokine receptor
Th17: T helper 17 cells
IMV: Invasive mechanical ventilation
LIS: Lung injury score
IDMC: Independent Data Monitoring Committee
PCR: Polymerase chain reaction
GHU: Groupe Hospitalier Universitaire
WHO: World Health Organization
PBW: Predicted body weight
RASS: Richmond Assessment Sedation Score
CT: Computed tomography
CAM-ICU: Confusion Assessment Method in ICU
MRC: Medical Research Council
REDCap: Research Electronic Data Capture
PaO_2_: Arterial partial pressure oxygen
FiO_2_: Inhaled oxygen fraction
PaO_2_/FiO_2_: Arterial partial pressure oxygen-inhaled oxygen fraction ratio
PEEP: Positive end-expiratory pressure
SAPS: Simplified Acute Physiology Score
KDIGO: Kidney Disease: Improving Global Outcomes
SAP: Statistical analysis plan
ITT: Intent-to-treat
mITT: Modified intent-to-treat
PP: Per protocol
SP: Safety population
IDMC: Independent Data Monitoring Committee
